# Interactions of Fe, Mn, Zn, and Cd in Soil–Rice Systems: Implications for Reducing Cd Accumulation in Rice

**DOI:** 10.3390/toxics13080633

**Published:** 2025-07-28

**Authors:** Yan Zhang, Su Jiang, Han Wang, Linfei Yu, Chunfu Li, Liqun Ding, Guosheng Shao

**Affiliations:** 1 China National Rice Research Institute, Hangzhou 310006, China; 2 Agricultural Technology Extension Center of Kaihua County, Quzhou 324300, China

**Keywords:** cadmium, micronutrient, acidic and alkaline soils, translocation factor, correlation

## Abstract

Cadmium (Cd) contamination in rice (*Oryza sativa* L.) poses serious health risks for human, necessitating effective mitigation strategies. This study investigated the effects of Cd stress on iron (Fe), manganese (Mn), zinc (Zn), and Cd accumulation and translocation in rice varieties with high (MY46) or low (ZS97B) Cd accumulation capacities grown in acidic and alkaline soils. Results demonstrated that Cd stress significantly inhibited plant growth, reducing plant height, shoot biomass, and grain yield in both soil types. Cd accumulation increased in roots, shoots, and grains, while Fe, Mn, and Zn concentrations decreased markedly. Molecular analysis revealed upregulation of metal transporter genes (*OsIRT1*, *OsNRAMP1*, *OsNRAMP5*) and the vacuolar sequestration gene (*OsHMA3*) in roots under Cd exposure. The translocation factor (TF) values of Mn and Zn from root to shoot were reduced in acidic soils, whereas Mn and Zn TFs exhibited an increasing trend in alkaline soils despite Cd exposure. Furthermore, correlation analyses indicated Mn and Zn play crucial roles in suppressing Cd accumulation in both acidic and alkaline soils. These findings provide critical insights for developing soil-specific strategies to reduce Cd accumulation in rice through micronutrient management.

## 1. Introduction

Cadmium (Cd), a non-essential heavy metal, has become prevalent in agricultural soils and food crops due to both natural processes and anthropogenic activities, including in southern China [1]. Cd contamination can disrupt key physiological processes in plants, including photosynthesis, mineral nutrient uptake, electron transport chain activity, and impair both vegetative growth and reproductive development through interference with phytohormonal regulation and metabolic pathways [2,3]. As one of the predominant staple crops in China, rice serves as the principal dietary vector for human Cd exposure, contributing 56% of the total estimated daily Cd intake within the general population [4]. More seriously, Cd can accumulate in the human body through the food chain, posing a serious threat to human health (e.g., Itai-itai disease) and food security [5]. Therefore, it is urgent to take effective strategies to reduce Cd accumulation in rice.

Research has demonstrated that key micronutrients (Fe, Mn, Zn) can effectively mitigate Cd uptake in rice; however, these findings are largely based on acidic soil conditions, while their interactions and efficacy in alkaline environments require further investigation. Although exogenous application of micronutrients (Fe, Mn, Zn) shows potential for regulating Cd accumulation and alleviating phytotoxicity in rice, empirical studies often yield contradictory results. Exogenous Fe application promotes the formation of iron plaque (primarily composed of ferrihydrite and goethite) on rice root surfaces, which effectively immobilizes Cd through specific adsorption and coprecipitation mechanisms [6], thereby creating a physicochemical barrier against Cd uptake and leading to a significant reduction in Cd accumulation in rice. However, the magnitude of this mitigating effect is closely related to soil pH, rice varieties, and Fe addition dosage [7,8]. It has also been found that there is a significant positive correlation between Cd sequestration in iron plaque and Cd accumulation in rice roots. Thus, Fe application may also promote Cd accumulation in rice [9]. Furthermore, exogenous Fe application reduces Cd accumulation in rice through Fe^2+^-mediated suppression of iron-regulated transporters (such as OsIRT1, OsNRAMP1), thereby limiting Cd uptake [10]. In addition, Mn fertilizers exhibit significant potential to mitigate Cd accumulation in rice [11,12], not only through the high adsorption affinity of manganese oxides for Cd but also via competitive inhibition between Mn^2+^ and Cd^2+^ at shared ion channels, thereby collectively reducing Cd bioavailability and limiting plant uptake [13,14,15]. However, studies have indicated that the effectiveness of exogenous Mn in reducing Cd accumulation in rice depend on its chemical form and application method [16]. Notably, soil application of MnSO_4_ was found to increase Cd uptake, while EDTA·Na_2_Mn significantly reduced it. Conversely, foliar application of either MnSO_4_ or EDTA·Na_2_Mn reduced Cd accumulation in grains but increased it in shoots. Similarly, the relationship between the trace elements Zn and Cd is also highly complex. Studies have shown that Zn application generally reduces Cd uptake and inhibits its translocation in rice plants [17,18]. However, others indicated that Cd accumulation in grains may increase under high soil Zn concentrations, particularly with excessive Zn supply [19,20]. Moreover, it was suggested that the varying effects of Zn supply on Cd accumulation in rice may be linked to soil pH and the ratio of Cd:Zn [21]. It has been found that in the presence of heavy metals, plants frequently show changed absorption pathways for vital nutrients, which may minimize or exacerbate the harmful impacts of Cd [22]. Accordingly, the relationship between these micronutrients and the heavy metal Cd is complex, and their interaction mechanisms are further influenced by soil conditions, rice cultivars, and other factors.

It is widely recognized that Fe, Mn, and Zn reduce Cd accumulation in rice primarily through competitive inhibition of divalent metal transporters, which are physiologically responsible for essential nutrient uptake. Specifically, OsNRAMP5, which belongs to the natural resistance-associated macrophage protein family, was reported as a major transporter for the uptake of Mn^2+^ and Cd^2+^ from the external soil solution into rice roots and also makes a small contribution to Fe^2+^ uptake [23]. OsNRAMP1, another transporter protein in the same family as OsNRAMP5, participates in the cellular uptake of Fe^2+^ and Cd^2+^ and is significantly upregulated by Fe deficiency [24,25]. In addition, iron-regulated transporter-like proteins (OsIRT1 and OsIRT2) also have been reported to participate in the cellular uptake of Fe^2+^ and Cd^2+^, and their expression levels are closely related to environmental iron content [26]. OsHMA2 and OsHMA3 (heavy-metal P-type ATPases) are responsible for Zn^2+^ and Cd^2+^ distribution through distinct subcellular mechanisms. The tonoplast-localized OsHMA3 mediates vacuolar sequestration of excess Cd^2+^, effectively restricting root-to-shoot translocation through cytoplasmic concentration buffering [27,28]. Conversely, the plasma membrane-bound OsHMA2 facilitates xylem loading of these divalent cations, thereby governing their systemic distribution to aerial tissues [29]. Moreover, the phloem-localized transporter OsLCT1 (low-affinity cation transporter) has been reported to be involved in mediating Cd^2+^ translocation to rice grains [30]. In addition to Cd^2+^, OsLCT1 also has the ability to transport K^+^, Ca^2+^, Mg^2+^, and Mn^2+^. Beyond the transporters previously characterized, multiple gene families, including OsZIP1, OsZIP5, OsZIP7, OsZIP9 (ZRT/IRT-like proteins), OsABCG36 (ATP-binding cassette transporter), OsCd1 (major facilitator superfamily), and OsFWL4 (membrane protein family), have been functionally validated to mediate Cd^2+^ uptake, xylem loading, and intercellular redistribution in rice [31,32,33,34,35,36].

To systematically elucidate the interaction mechanisms among Fe, Mn, Zn, and Cd, we investigated two rice genotypes with contrasting Cd-accumulation characteristics grown in different soil types (acidic vs. alkaline). The dynamics of Fe, Mn, Zn, and Cd across different soil types, along with their variations in root-to-grain translocation efficiency and accumulation patterns in rice, were systematically characterized. Differences in expression patterns of Fe, Mn, Zn, and Cd relevant key transporter genes were also evaluated. Our results thus provide the fundamental information to reduce Cd accumulation in rice by utilizing Fe, Mn, and Zn micronutrients.

## 2. Materials and Methods

### 2.1. Experimental Materials

A glasshouse pot experiment was conducted in Hangzhou city (119°57′ E, 30°03′ N), Zhejiang Province, China, with the exposure of natural light. Two kinds of soil with different acidity and alkalinity were collected from paddy fields in Quzhou city, Zhejiang Province. The basic physicochemical properties and nutrient levels of acidic soil (labeled “ACS”) and alkaline soil (labeled “ALS”) are detailed in Supplementary Table S1. In addition, two indica genotypes, Miyang 46 (labeled “MY46”, high-Cd accumulated) and Zhenshan 97B (labeled “ZS97B”, low-Cd accumulated), were used in this experiment.

### 2.2. Treatment Condition and Sample Collection

Two treatments were set for each soil and genotype: control group (Cd 0, no exogenous Cd) and treatment group (Cd 1, 1 mg.kg^−1^ Cd, supplied by CdSO_4_.8/3 H_2_O). Soils were air-dried and disaggregated. Ten kg of each type of soil was filled into a pot with a drainage hole at the bottom, and exogenous Cd was added into the treatment group pots and aged for 3 months at 80% of water-holding capacity before transplanting. The experiment used a Randomized Complete Block Design (RCBD) with 3 blocks (corresponding to 3 replicates). Within each block, the factorial combinations of 2 plant varieties × 2 soil conditions × 2 treatments were randomly assigned to experimental units. Fifteen-day-old uniform seedlings of MY46 and ZS97B were selected and transplanted to corresponding pots that had been amended with base nutrients. Each pot contained nine rice seedlings transplanted into three holes.

At the rice tillering and maturity stages, rice tissues (roots, shoots, and grains) of MY46 and ZS97B, as well as soil samples, were collected. Root samples for gene expression at tillering stage were frozen immediately with liquid nitrogen and refrigerated at −80 °C. Samples of plant tissue used to detect the contents of elements were dried at 105 °C for 30 min, then dried for 72 h at 60 °C till a constant weight was achieved. Soil samples were kept indoors for natural air-drying.

### 2.3. Element Content Determination

Root and shoot samples (0.25 g) or grain samples (0.5 g) were subjected to overnight digestion with 8 mL HNO_3_ at room temperature. The samples were then transferred to a graphite digestion system (ZEROM ProD60, Changsha Zerom Instrument and Meter Co., Ltd., Changsha, China) and heated at 140 °C for 1.5 h. After brief cooling, 2 mL H_2_O_2_ was added, followed by continued heating at 140 °C for an additional hour. After cooling, the final solution was made up to 50 mL by the addition of Milli-Q water (Millipore Filter Co., Bedford, MA, USA). The reagent blanks were treated in the same way as the samples. Concentrations of Fe, Mn, Zn, and Cd in the samples were measured using ICP-OES (Thermo-Jarrell Ashe, Franklin, MA, USA) under 1300 W radiofrequency power, 15 L min^−1^ plasma gas flowrate, 0.2 L min^−1^ auxiliary gas flow rate, and 0.8 L min^−1^ nebulizer gas flow rate. From the axial plasma view, the emission lines (nm) were chosen as follows: Fe (II) 238.204, Mn (II) 257.610, Zn (II) 206.200, and Cd (II) 228.802.

### 2.4. Measurement of Soil pH, Available Metal Concentration

Soil pH was measured on a water-to-soil ratio of 2.5:1 suspension by using a digital pH meter (FiveGo F2, Mettler Toledo, Greifensee, Switzerland). The bioavailable trace elements in the soil were extracted using 0.005 mol/L DTPA (pH 7.3) at a soil-to-solution ratio of 1:2.5 (*w*/*v*). The mixture was agitated for 2 h at room temperature. The extracts were filtered with a 0.45 µm filter and then analyzed for element concentration by using ICP-OES.

### 2.5. Gene Expression Analysis

Total RNAs were extracted from the roots of MY46 and ZS97B at the maximum tillering stage of rice by using the TRIzol reagent (Aidlab Biotech, Beijing, China) according to the manufacture’s instruction. The complementary DNA (cDNA) was synthesized by using the MonScriptTM RTIII Super Mix with dsDNAase Kit with gDNA Eraser (Perfect Real Time) (Monad Biotech Co., Ltd., Suzhou, China). qRT-PCR was performed with MonAmpTM SYBR^®^ Green qPCR Mix (Monad Biotech Co., Ltd., Suzhou, China) in a total volume of 20 μL. The details of the primers for target genes and the internal control gene (*OsActin*) are provided in the Supplementary Materials. Each cDNA sample was run in triplicate with three biological replicates. Data were analyzed with 2^−ΔΔCT^ method as previously reported [37].

### 2.6. Statistical Analysis

All statistical analyses were carried out by using R software (version 4.4.2). Values were presented as means ± SD (*n* = 3), and significant differences were determined by the Fisher’s least significant difference (LSD) test at *p* < 0.05. Pearson correlation analysis was used to analyze the relationship between the contents of Fe, Mn, Zn, and Cd in soil and plants. Data were plotted by Excel 2024 and R software.

## 3. Results

### 3.1. Agronomic Traits and Accumulation of Fe, Mn, Zn, and Cd in Rice

We analyzed plant height, shoot dry weight, SPAD value, and 1000-grain weight in two rice genotypes (MY46 and ZS97B) at both tillering and maturity stages separately. At tillering stage, Cd exposure did not significantly affect plant height in acidic soils for either genotype (MY46 or ZS97B). However, in alkaline soil, MY46 showed a significant 4.43% reduction in plant height, while ZS97B remained unaffected. Cd stress significantly decreased shoot dry weight in both cultivars across soil types. Furthermore, Cd exposure significantly reduced rice SPAD values, except for MY46 in acidic soil (Table S2). Notably, it demonstrated significant Cd-induced growth inhibition across all measured parameters at maturity stage in both genotypes (Table S3).

**Figure 1:**
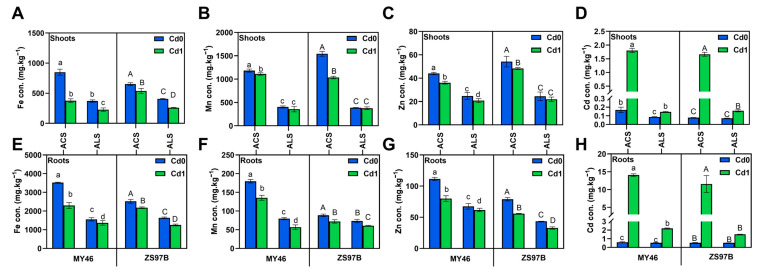
Concentrations of Fe (**A**,**E**), Mn (**B**,**F**), Zn (**C**,**G**), and Cd (**D**,**H**) in shoots and roots of MY46 and ZS97B rice plants under varying Cd treatments in acidic and alkaline soils at the maximum tillering stage. ACS, acidic soil; ALS, alkaline soil; Cd 0, no Cd stress; Cd 1, add 1 mg/kg Cd stress. Data are means ± SD (*n* = 3). Means with different lowercase (MY46) and capital (ZS97B) letters are significantly different (LSD, *p* < 0.05) with regard to treatments.

To assess the effects of Cd on trace element accumulation in rice grown in acidic and alkaline soils, concentrations of Fe, Mn, Zn, and Cd were analyzed in plants at tillering and maturity stages (Figure 1 and Figure S1). Consistently, rice grown in alkaline soil exhibited lower trace element (Fe, Mn, Zn, and Cd) concentrations in roots and shoots compared to acidic soil across all growth stages, regardless of Cd exposure. Root Cd concentrations at the tillering stage showed significant increases under Cd stress. In acidic soil, root Cd levels in MY46 and ZS97B were 23.90-fold and 21.79-fold higher than their respective controls, significantly higher than the 4.19- and 2.89-fold increases observed in alkaline soil (Figure 1H). Contrasting with Cd accumulation, root Fe, Mn, and Zn concentrations displayed significant reductions under Cd exposure (Figure 1). In acidic soil, Fe decreased by 34.84% (MY46) and 13.78% (ZS97B), Mn by 24.67% and 18.52%, and Zn by 28.19% and 29.34% relative to controls. Under alkaline conditions, Fe decreased by 12.20% (MY46) and 23.53% (ZS97B), Mn by 28.73% and 17.10%, and Zn by 8.67% and 24.41%. Shoot tissues also exhibited parallel trends in Fe, Mn, Zn, and Cd concentrations under both soil conditions. These characteristics of the changes described above even persisted into rice maturity (Figure S1). In acidic soil, grain Cd reached 25.12-fold (MY46) and 8.78-fold (ZS97B) of controls, significantly higher than the respective 2.89- and 2.50-fold increases observed in alkaline soil (Figure S2D). Fe, Mn, and Zn concentrations decreased differentially across soils, with MY46 showing 13.71–36.86% reductions in acidic soil and 2.09–32.67% in alkaline conditions, while ZS97B exhibited 8.81–29.18% and 11.59–14.63% decreases, respectively (Figure S2A–C). In summary, Cd can reduce the accumulation of Fe, Mn and Zn in rice, which is closely related to genotype and soil type.

### 3.2. Translocation of Fe, Mn, Zn, and Cd in Rice

Furthermore, the root-to-shoot (TF_s/r_) and shoot-to-grain (TF_b/s_) translocation factors were applied to quantitatively evaluate the translocation patterns of Fe, Mn, Zn, and Cd in two rice genotypes (MY46 and ZS97B) across soil types and Cd levels. At the tillering stage, TF_s/r_ values of Cd and Fe (<1) suggested preferential accumulation in roots, whereas Mn exhibited shoot enrichment (TF_s/r_ > 1) (Table 1). Under Cd exposure, TF_s/r_ of Fe and Cd in both genotypes decreased significantly in acidic and alkaline soils versus controls, with MY46 showing greater sensitivity. TF_s/r_ of Mn and Zn decreased significantly in acidic soil but increased markedly in alkaline soil across both varieties. Compared to the tillering stage, TF_s/r_ of Fe at maturity were consistently lower across soil types and genotypes, while Mn and Zn translocation patterns mirrored tillering-stage trends. Notably, TF_s/r_ of Cd displayed soil-dependent divergence: increased in acidic soil (1.13- to 2.42-fold) but decreased in alkaline soil (61.54% to 53.85%) under Cd exposure. At the maturity stage, exogenous Cd universally elevated the TF_b/s_ of Zn in both genotypes, irrespective of soil type. The TF_b/s_ of Cd exhibited divergent responses to Cd stress between soil types: significantly decreased in acidic soil but increased in alkaline soil, regardless of rice genotype. Thus, Cd significantly influences the translocation efficiency of Fe, Mn, and Zn across rice growth stages, and relevant variations were modulated by soil type and rice genotype.

### 3.3. Availability of Fe, Mn, Zn, and Cd in Soils

**Figure 2:**
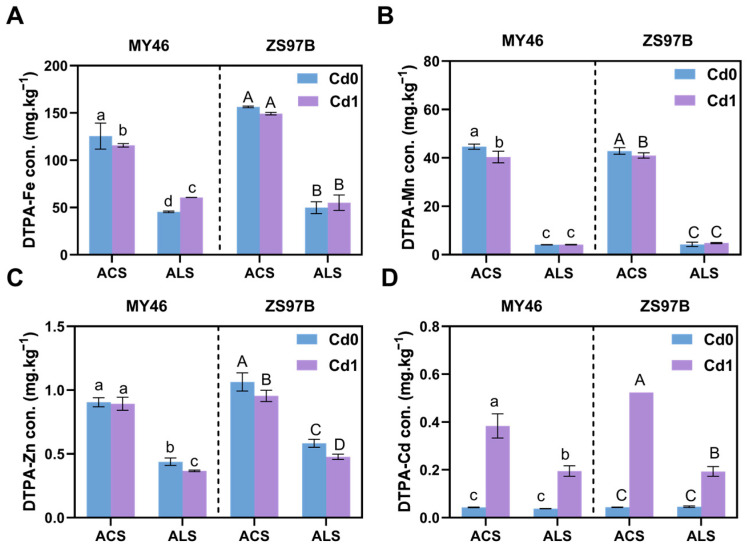
Concentrations of DTPA-extractable Fe, Mn, Zn, and Cd in acidic and alkaline soils under varying Cd stresses during the maximum tillering stage of rice. (**A**–**D**) Available contents of Fe, Mn, Zn, and Cd were determined by DTPA extractor. ACS, acidic soil; ALS, alkaline soil; Cd 0, no Cd stress; Cd 1, add 1 mg/kg Cd stress. Data are means ± SD (*n* = 3). Means with different lowercase (MY46) and capital (ZS97B) letters are significantly different (LSD, *p* < 0.05) with regard to treatments. The dotted lines separate the two varieties.

Compared with the control, exogenous Cd application at the tillering stage reduced soil pH in both acidic (MY46: −0.12 units; ZS97B: −0.08 units) and alkaline soils (MY46: −0.39 units; ZS97B: −0.29 units), with alkaline soils showing significantly greater reductions in both varieties. At maturity, only MY46 exhibited significant decreases under both soil conditions, whereas ZS97B showed slight but non-significant decreases (Figure S3). In general, the content of available Fe, Mn, Zn, and Cd was all higher in acidic soil than in alkaline soil (Figure 2 and Figure S4), no matter at the maximum tillering or maturity stage, and concentration of available Cd in both soils increased significantly under Cd stress. At the seedling stage, DTPA-extractable Fe (DTPA-Fe) concentration in acidic soil decreased significantly by 7.83% (MY46) and 4.83% (ZS97B) compared to the control, whereas in alkaline soil, it increased by 33.47% (MY46) and 10.45% (ZS97B). The tendency of DTPA-extractable Mn (DTPA-Mn) was the same as DTPA-Fe in the soils, but it was not significant in alkaline soil. DTPA-extractable Zn (DTPA-Zn) concentrations declined in both soils, with reductions of 1.37% (MY46) and 10.25% (ZS97B) in acidic soil and more pronounced decreases of 16.41% (MY46) and 18.10% (ZS97B) in alkaline soil compared to the control. The trend of maturity was similar to that of tillering, except for the decreases of DTPA-Fe and DTPA-Mn in alkaline soil at the maturity stage of rice (Figure S4). As a result, Cd has an important effect on the availability of Fe, Mn, and Zn in soil.

### 3.4. Expression Patterns of Transporter Genes in the Roots

**Figure 3:**
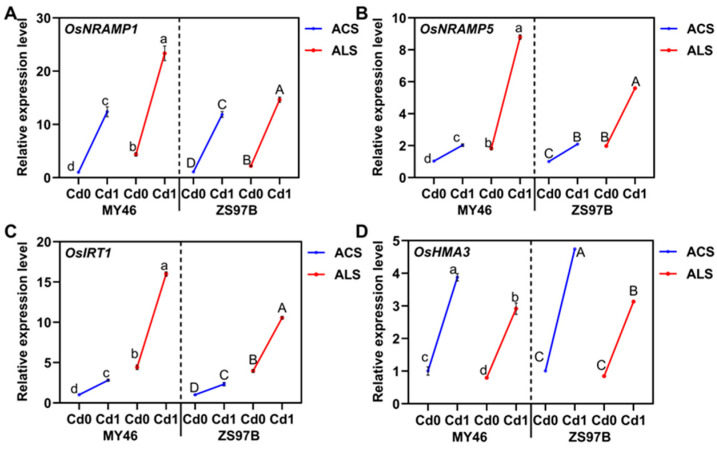
The relative expression levels of *OsNRAMP1* (**A**), *OsNRAMP5* (**B**), *OsIRT1* (**C**), and *OsHMA3* (**D**) in roots of MY46and ZS97B under varying Cd treatments in acidic and alkaline soils at the maximum tillering stage, with OsACTIN as the reference gene. Primers are listed in supporting information Table S4. ACS, acidic soil; ALS, alkaline soil; Cd 0, no Cd stress; Cd 1, add 1 mg/kg Cd stress. Data are means ± SD (*n* = 3). Means with different lowercase (MY46) and capital (ZS97B) letters are significantly different (LSD, *p* < 0.05) with regard to treatments. The dotted lines separate the two varieties.

Fe, Mn, Zn, and Cd accumulation were also associated with differential expression patterns of metal transporters. Therefore, we analyzed the root expression profiles of *OsNRAMP1*, *OsNRAMP5*, *OsIRT1*, and *OsHMA3* in two rice genotypes (MY46 and ZS7B) grown in Cd-contaminated acidic and alkaline soils during the tillering stage. As shown, root expression of *OsNRAMP1*, *OsNRAMP5*, and *OsIRT1* was higher in alkaline soil than acidic soil across genotypes, except for *OsHMA3*, with these trends being Cd-independent (Figure 3). Cd exposure significantly induced upregulation of *OsNRAMP1*, *OsNRAMP5*, *OsIRT1*, and *OsHMA3* in both MY46 and ZS97B across soil types. Comparative analysis revealed differential expression magnitudes under acidic versus alkaline conditions. *OsNRAMP5* exhibited 1.97- to 2.10-fold increases in acidic soil and 4.84- to 2.83-fold elevations in alkaline soil. *OsNRAMP1* demonstrated the most pronounced induction, with 12.37- to 10.85-fold increments in acidic soil and 5.35- to 6.65-fold enhancements in alkaline soil. *OsIRT1* showed upregulation ranging from 2.81- to 2.32-fold in acidic soil and 3.61- to 2.67-fold in alkaline soil. OsHMA3 expression increased consistently, with 3.86- to 4.72-fold in acidic soil and 3.65- to 3.69-fold in alkaline soil. Notably, while the four genes displayed congruent induction patterns between genotypes, their relative expression amplitudes varied depending on soil conditions.

### 3.5. Correlations of Fe, Mn, Zn, and Cd in Plants with Extractable Concentrations in Soils

**Figure 4:**
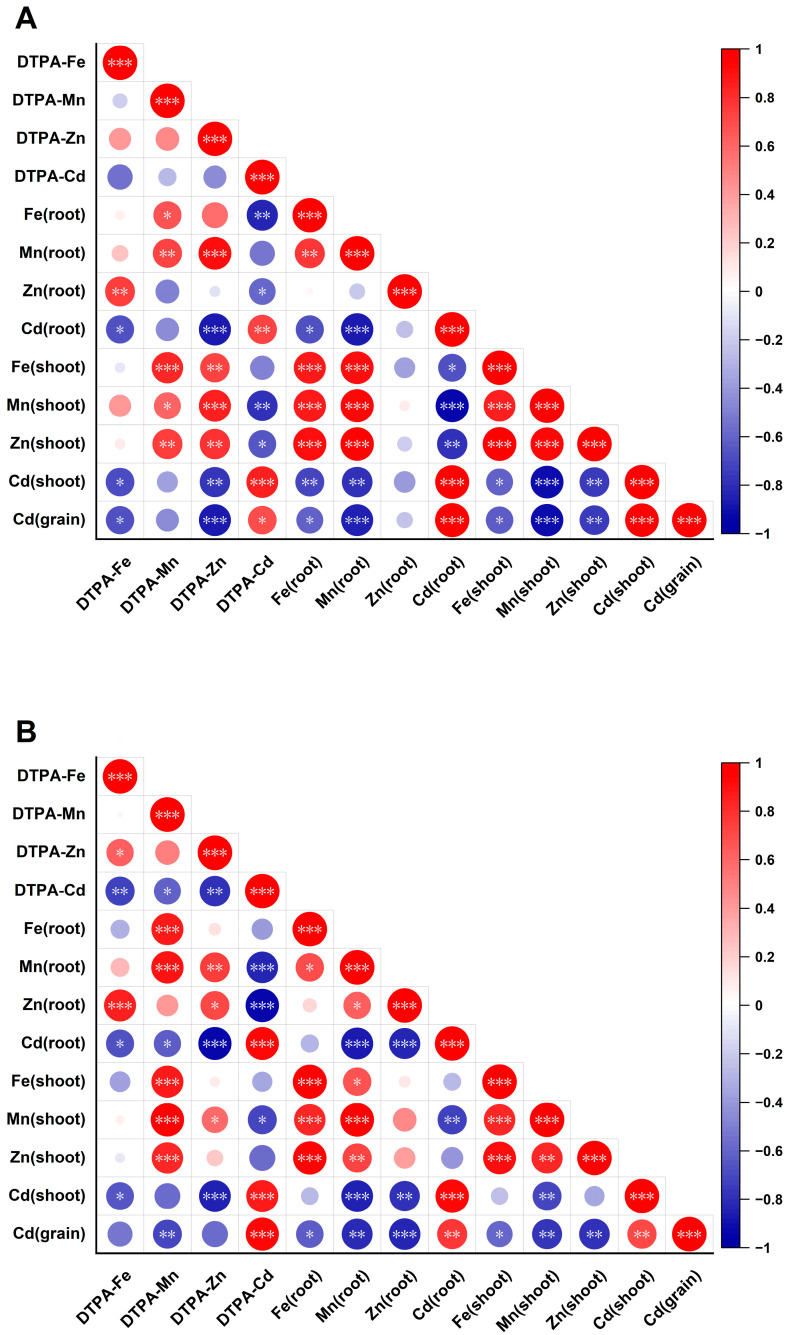
Heatmap and Pearson correlations of Fe, Mn, Zn, and Cd concentrations in rice tissues and DTPA-extractable in acidic (**A**) and alkaline (**B**) soils at rice maturity stage. *, *p* < 0.05; **, *p* < 0.01; ***, *p* < 0.001. The size of the circles represents the strength of correlation (larger size = higher absolute correlation coefficient.

At rice maturity stage, significant correlations (*p* < 0.01) were observed between DTPA-extractable Fe, Mn, Zn, and Cd in soils and their concentrations in rice tissues (Figure 4). DTPA-extractable Fe, Mn, and Zn exhibited negative correlations with DTPA-extractable Cd in both acidic and alkaline soils, with the strongest associations in alkaline soil (Fe: *r* = −0.73; Zn: *r* = −0.79). DTPA-extractable Cd exhibited significant positive correlations with Cd accumulation in rice roots, shoots, and grains across both acidic and alkaline soil conditions. In acidic soil, root Mn showed a significant negative correlation with root Cd (*r* = −0.87). Root Fe and Mn exhibited positive correlations with shoot Fe, Mn, and Zn but displayed inverse associations with shoot Cd (Fe: *r* = −0.71; Mn: *r* = −0.79). Notably, grain Cd showed strong negative correlations with DTPA-Zn (*r* = −0.87), root Mn (*r* = −0.86), shoot Mn (*r* = −0.90), and shoot Zn (*r* = −0.75). In alkaline soil, DTPA-Mn showed significantly negative correlation with grain Cd (*r* = −0.71). Root Mn and Zn demonstrated consistent negative correlations with Cd accumulation in roots (Mn: *r* = −0.87; Zn: *r* = −0.83), shoots (Mn: *r* = −0.85; Zn: *r* = −0.78), and grains (Mn: *r* = −0.81; Zn: *r* = −0.84). Shoot Mn and Zn also showed significant negative correlation with grain Cd (Mn: *r* = −0.76; Zn: *r* = −0.80). These distinct correlation patterns demonstrate that Cd translocation in the soil–rice system is primarily governed by Fe, Mn, and Zn homeostasis, with Mn and Zn playing particularly pivotal roles.

## 4. Discussion

Strategies for nutrient management, particularly the application of trace elements, to reduce Cd accumulation in rice have been extensively studied, though the findings have demonstrated significant variability between studies. In the present study, we systematically investigated the mechanistic interactions between trace elements (Fe, Mn, Zn) and Cd within soil–plant systems, employing a comparative analysis of two rice cultivars (MY46 vs. ZS97B) and contrasting soil types (acidic vs. alkaline soil). Fe, Mn, and Zn concentrations in rice plants grown in both acidic and alkaline soils were all significantly reduced accompanied by the exogenous Cd, while concurrently enhancing Cd accumulation. It mainly attributed to the antagonistic competition of Cd^2+^ with Fe^2+^, Mn^2+^, and Zn^2+^ for binding sites on divalent metal transporters localized in rice root membranes, thereby suppressing the uptake of these essential micronutrients [23,24,25,26,38]. The conserved upregulation of metal transport genes (*OsIRT1*, *OsNRAMP1*, *OsNRAMP5*) and vacuolar Cd sequestration gene (*OsHMA3*) was observed in the roots of both rice varieties under Cd stress across divergent soil types. The altered expression of these genes partially reflects the competitive interactions between essential micronutrients (Fe, Mn, Zn) and the toxic heavy metal Cd. Notably, previous studies have demonstrated that these metal transporter genes show differential induction patterns under micronutrient stress, e.g., OsNRAMP1 responds to both Fe and Mn deficiency [39], OsNRAMP5 is activated by Fe and Zn deficiency [40,41], while OsIRT1 exhibits specific upregulation under Fe-deficient conditions [42,43]. Furthermore, the lower expression levels of the *OsNRAMP1*, *OsNRAMP5*, and *OsIRT1* in rice roots under acidic soil conditions may result from higher availability of Fe, Mn, and Zn [44], which suppresses the induction of these metal transporter genes under micronutrient-sufficient conditions [24,45].

TF is an effective indicator for evaluating the mobility of elements between different plant tissues. At the seedling stage, exogenous Cd addition reduced the translocation of Fe, Mn, Zn, and Cd from roots to shoots in acidic soils. This phenomenon may be attributed to Cd-induced protective mechanisms, such as vacuolar sequestration mediated by OsHMA3 upregulation, which confines Cd to root cell vacuoles and limits its translocation [27]. Notably, our study observed a significant increase in *OsHMA3* expression across different rice varieties under Cd stress. Additionally, Cd competitively inhibits the uptake of Fe, Mn, and Zn by binding to their respective transport proteins (e.g., OsIRT1, OsNRAMP5, and OsZIPs) [26,40,45], thereby suppressing root-to-shoot translocation of these essential micronutrients. In contrast, rice grown in alkaline soil exhibited enhanced root-to-shoot translocation of Mn and Zn under Cd stress, consistent with previous studies [46,47]. The root-to-shoot transfer patterns at maturity were similar to those at the seedling stage. Under Cd stress, shoot-to-grain Zn translocation increased, whereas Cd translocation varied with soil types. Specifically, Cd transfer capacity decreased significantly in acidic soil but increased markedly in alkaline soil. This suggests that rice may mitigate Cd toxicity by preferentially allocating Zn to grains [48]. On the other hand, under alkaline conditions, the nutrient content in rice is significantly lower than in acidic soils. To maintain ion homeostasis, the expression of trace element and Cd cotransporters was upregulated, reaching significantly higher levels than in acidic soils, which likely contributed to the enhanced translocation of both Zn and Cd. Overall, the mutual influence of Fe, Mn, Zn, and Cd is reflected in their transfer patterns, which are closely tied to soil conditions. Future studies should be conducted to explore the key environmental factors that contribute to these patterns.

With the increase of exogenous Cd, the fraction of the bioavailable Cd in both acidic and alkaline soils has been significantly enhanced. In acidic soils, the enhanced Cd bioavailability may be attributed to both increased Cd solubility and its stronger adsorption competition under reduced Eh, which promotes Cd^2+^ dominance over Fe^2+^, Mn^2+^, and Zn^2+^ [49]. Consequently, the available concentrations of Fe, Mn, and Zn were consistently reduced, consistent with the previous research findings [50]. In alkaline soils, available Fe and Mn increased, while available Zn decreased significantly. This is similar to the previously reported result that under alkaline conditions (pH 7.8), Cd stress led to a 1.5- to 2-times increase in the concentration of Mn^2+^ in the rhizosphere of rice [51]. This may be attributed to exogenous Cd addition reducing soil pH via competitive adsorption of Cd^2+^ and H^+^ on soil particle surfaces [52,53]. The resulting decrease in pH promotes the dissolution of Fe and Mn oxides/hydroxides, which exist primarily in these forms in alkaline soils [54]. In contrast, despite their geochemical similarity, Cd^2+^ adsorbs more strongly to soil colloids than Zn^2+^, displacing Zn^2+^ into the liquid phase or forming insoluble precipitates (e.g., Zn-Cd carbonates) [50,55]. This demonstrates that the interactions between Cd and trace elements (Fe, Mn, Zn) vary significantly and are highly dependent on soil conditions.

To evaluate the relationships between Cd accumulation in rice grains and the distribution of key elements in soil, roots, and shoots, we performed correlation analyses across different soil conditions. In this study, rice grain Cd content is significantly correlated with soil-available Cd, root Cd, and shoot Cd, irrespective of acidic or alkaline soil. Under acidic soil conditions, DTPA-Zn, root Mn, shoot Mn, and shoot Zn exhibited strongly significant negative correlations with grain Cd. It suggests that increasing rice root Mn and Zn concentrations can reduce the rice grain Cd accumulation under acidic soil. In addition, DTPA-Mn, root Mn, root Zn, shoot Mn, and shoot Zn all showed strongly significant negative correlations with grain Cd under alkaline conditions. Compared with Mn and Zn, Fe has a relatively small effect on inhibiting Cd accumulation in rice in this study. This might be because plants have evolved two Fe acquisition strategies to maintain a relatively stable absorption efficiency in soils with different soil conditions [56,57,58], as well as the corresponding transcriptional protein levels. Additionally, available Zn dominantly controls Cd accumulation in rice in acidic soils, whereas in alkaline soils, available Mn becomes the primary regulator. This is mainly due to the high mobility of Zn^2+^ and its competitive interactions with Cd^2+^ for binding sites [43]. In contrast, in alkaline soils, available Mn becomes the dominant regulator, as increased pH reduces Zn^2+^ solubility and promotes Mn oxide formation, thereby enhancing Cd adsorption and suppressing Cd activity [59]. Overall, these findings suggest increasing Mn and Zn concentrations in the root system and leaves effectively reduces Cd accumulation in rice grains under acidic or alkaline soil conditions with potentially different patterns.

## 5. Conclusions

This study elucidates the complex interactions between essential micronutrients (Fe, Mn, and Zn) and Cd dynamics in soil–rice systems. Our results demonstrate that these micronutrients consistently exhibit antagonistic effects against Cd bioavailability throughout the soil–plant continuum and across both rice growth stages. Notably, Mn and Zn serves as the primary inhibitors of Cd accumulation in acidic and alkaline soils. These findings establish a mechanistic framework for developing targeted mitigation strategies, including optimized trace element fertilization and Mn/Zn-enriched soil amendments, to effectively reduce Cd contamination in rice production systems.

## Figures and Tables

**Table 1:** Translocation factors (TFs) of Fe, Mn, Zn, and Cd in rice under different growth stages and treatments.

Rice Varieties	Types of Soil	Treatments	Tillering Stage				Maturity Stage							
			Fe_TFs/r_	Mn_TFs/r_	Zn_TFs/r_	Cd_TFs/r_	Fe_TFs/r_	Mn_TFs/r_	Zn_TFs/r_	Cd_TFs/r_	Fe_TFb/s_	Mn_TFb/s_	Zn_TFb/s_	Cd_TFb/s_
MY46	ACS	Cd0	0.24 ± 0.01 a	8.21 ± 0.37 a	0.45 ± 0.02 a	0.29 ± 0.02 a	0.04 ± 0.00 c	11.76 ± 0.58 a	1.11 ± 0.01 a	0.07 ± 0.02 c	0.03 ± 0.00 b	0.02 ± 0.00 c	0.41 ± 0.04 d	0.65 ± 0.16 a
		Cd1	0.16 ± 0.02 b	6.58 ± 0.25 b	0.40 ± 0.01 b	0.13 ± 0.00 c	0.04 ± 0.00 c	9.58 ± 0.36 b	0.94 ± 0.06 b	0.17 ± 0.02 a	0.03 ± 0.00 b	0.02 ± 0.00 c	0.56 ± 0.01 c	0.40 ± 0.03 b
	ALS	Cd0	0.24 ± 0.02 a	5.07 ± 0.28 c	0.34 ± 0.03 bc	0.17 ± 0.01 b	0.11 ± 0.01 b	3.49 ± 0.06 c	0.63 ± 0.05 c	0.13 ± 0.00 b	0.04 ± 0.00 a	0.04 ± 0.00 a	0.73 ± 0.04 b	0.38 ± 0.03 b
		Cd1	0.17 ± 0.03 b	6.27 ± 0.60 b	0.37 ± 0.04 c	0.07 ± 0.00 d	0.16 ± 0.01 a	4.67 ± 0.25 d	0.81 ± 0.14 b	0.08 ± 0.00 c	0.02 ± 0.00 c	0.03 ± 0.00 b	1.12 ± 0.15 a	0.67 ± 0.01 a
ZS97B	ACS	Cd0	0.26 ± 0.01 A	17.41 ± 0.42 A	0.87 ± 0.01 A	0.15 ± 0.01 A	0.05 ± 0.00 C	11.95 ± 0.06 A	2.91 ± 0.20 A	0.24 ± 0.03 A	0.02 ± 0.00 B	0.02 ± 0.00 C	0.30 ± 0.01 C	0.38 ± 0.03 A
		Cd1	0.25 ± 0.02 A	14.35 ± 0.55 B	0.69 ± 0.02 B	0.15 ± 0.04 A	0.06 ± 0.00 A	9.60 ± 0.12 B	1.90 ± 0.11 B	0.27 ± 0.02 A	0.02 ± 0.00 B	0.02 ± 0.00 C	0.51 ± 0.05 B	0.26 ± 0.02 B
	ALS	Cd0	0.25 ± 0.00 A	5.30 ± 0.25 D	0.56 ± 0.06 C	0.14 ± 0.00 AB	0.04 ± 0.00 D	3.00 ± 0.06 D	1.24 ± 0.08 C	0.13 ± 0.01 B	0.04 ± 0.00 A	0.03 ± 0.00 B	0.52 ± 0.03 B	0.29 ± 0.02 B
		Cd1	0.21 ± 0.01 B	6.20 ± 0.24 C	0.67 ± 0.01 B	0.11 ± 0.01 B	0.06 ± 0.00 B	4.89 ± 0.21 C	1.27 ± 0.13 C	0.07 ± 0.01 C	0.04 ± 0.00 A	0.04 ± 0.00 A	0.60 ± 0.04 A	0.36 ± 0.06 A

ACS, acidic soil; ALS, alkaline soil; Cd0, no Cd stress; Cd1, add 1 mg/kg Cd stress; TF_s_/_r_, root-to-shoot translocation factor; TF_b_/_s_, shoot-to-brown-rice translocation factor. Data are means ± SD (*n* = 3). Means with different lowercase (MY46) and capital (ZS97B) letters are significantly different (LSD, *p* < 0.05) with regard to treatments.

## Data Availability

The original contributions presented in this study are included in the article. Further inquiries can be directed to the corresponding authors.

## Supplementary Materials

The following supporting information can be downloaded at https://www.mdpi.com/article/10.3390/toxics13080633/s1. Table S1. Basic physicochemical properties and nutrient levels of the acidic (ACS) and alkaline (ALS) soils used in the study. Table S2. Agronomic traits of rice at the maximum tillering stage under varying cadmium stress in acidic and alkaline soils. Table S3. Agronomic traits of rice at the maturity stage under varying cadmium stress in acidic and alkaline soils. Table S4. Primers for gene expression assay. Figure S1. Concentrations of Fe (A,E), Mn (B,F), Zn (C,G), and Cd (D,H) in shoots and roots of MY46 and ZS97B rice plants under varying Cd treatments in acidic and alkaline soils at the maturity stage. Figure S2. Concentrations of Fe (A), Mn (B), Zn (C), and Cd (D) in brown rice of MY46 and ZS97B under varying Cd treatments in acidic and alkaline soils. Figure S3. Effects of cadmium stress on soil pH at rice tillering and maturity stages in acidic and alkaline soils. Figure S4. Concentrations of DTPA-extractable Fe, Mn, Zn and Cd in acidic and alkaline soils under varying cadmium stresses during the maturity stage of rice.
