# Supplementary material for: Interactions of Fe, Mn, Zn, and Cd in Soil–Rice Systems: Implications for Reducing Cd Accumulation in Rice
Source: Toxics. 2025 Jul 28;13(8):633. doi: 10.3390/toxics13080633 (PMC12390429; doi:10.3390/toxics13080633)
Supplement: Supplementary file 1 [file toxics-13-00633-s001.zip › toxics-3721035-supplementary.pdf]

Supplementary Materials

Table S1. Basic physicochemical properties and nutrient levels of the acidic (ACS) and alkaline (ALS) soils used in the study.

Types of soil	pH	OM g.kg^{-1}	CEC cmol.kg^{-1}	TN g.kg^{-1}	TP mg.kg^{-1}	TK g.kg^{-1}	Fe g.kg^{-1}	Mn mg.kg^{-1}	Zn mg.kg^{-1}	Cd mg.kg^{-1}
**ACS**	5.80± 0.03 b	35.60± 0.31 b	15.10± 0.27 b	1.70± 0.05 b	545.80± 13.20 b	1.69± 0.08 b	24.93± 0.46 b	324.55± 8.51 b	69.56± 1.43 b	0.16± 0.01 b
**ALS**	7.80± 0.03 a	44.40± 0.96 a	23.25± 1.52 a	2.30± 0.15 a	745.10± 15.01 a	1.92± 0.07 a	38.56± 0.60 a	417.61± 3.85 a	98.60± 1.55 a	0.29± 0.01 a

ACS, acidic soil; ALS, alkaline soil; OM, organic matter; EC, electrical conductivity; TN, total nitrogen; TP, total phosphorus; TK, total potassium. Means with different lowercase letters indicate statistically significant differences among soil types ($p < 0.05$).

Table S2. Agronomic traits of rice at the maximum tillering stage under varying cadmium stress in acidic and alkaline soils.

**Rice**	**Types of**	**Treatments**	**Plant height**	**Dry weight of shoots**	**SPAD value**
MY46	ACS	Cd0	55.56±1.16 a	11.62±0.66 a	41.26±1.76 b
		Cd1	54.30±1.30 ab	7.93±0.05 b	41.25±0.51 b
	ALS	Cd0	55.46±1.15 a	12.39±0.38 a	46.04±1.28 a
		Cd1	53.00±0.61 b	6.28±0.09 c	42.63±1.28 b
ZS97B	ACS	Cd0	71.78±1.01 B	15.02±0.07 A	47.38±1.09
		Cd1	69.83±1.26 B	10.45±0.21 C	44.15±0.68
	ALS	Cd0	74.52±3.30 A	15.59±0.47 A	49.83±1.19
		Cd1	74.72±1.04 A	11.77±0.35 B	44.40±1.09

ACS, acidic soil; ALS, alkaline soil; Cd0, no Cd stress; Cd1, add 1 mg/kg Cd stress; Data are means ± SD ($n = 3$). Mean with different lowercase (MY46) and capital (ZS97B) letters are significantly different (LSD, $p < 0.05$) with regard to treatments.

Table S3. Agronomic traits of rice at the maturity stage under varying cadmium stress in acidic and alkaline soils.

Rice varieties	Types of soil	Treatments	Plant height (cm)	Dry weight of shoots (g)	1000-grain weight (g)
MY46	ACS	Cd0	56.27±0.75 a	36.82±1.11 b	24.07±0.33 a
		Cd1	49.35±0.21 c	29.49±0.29 d	20.78±0.59 c
	ALS	Cd0	56.10±0.36 a	40.86±0.74 a	22.61±0.52 b
		Cd1	52.50±0.00 b	33.05±0.42 c	20.35±0.21 c
ZS97B	ACS	Cd0	72.55±0.64 B	29.39±0.36 C	23.39±0.64 A
		Cd1	68.50±0.50 C	27.75±1.00 C	22.11±0.92 AB
	ALS	Cd0	78.10±0.57 A	38.59±0.54 A	23.11±0.01 AB

	Cd1	73.90±0.57 B	32.29±0.83 B	21.89±0.32 B

ACS, acidic soil; ALS, alkaline soil; Cd0, no Cd stress; Cd1, add 1 mg/kg Cd stress; Data are means ± SD (n = 3). Mean with different lowercase (MY46) and capital (ZS97B) letters are significantly different (LSD, $p < 0.05$) with regard to treatments.

Table S4. Primers for gene expression assay.

Gene	Former primer (5'–3')	Reverse primer (5'–3')
OsHMA3	AGCCTCCAAGAAGCCAACTG	CAACATTTGGCAACGACGG
OsIRT1	CGATGTGCTTCCACCAGATG	GCGTCGTGGTGGAGAAGAAG
OsNRAMP1	GTGATTGCTTCCGATATTCCA	CAACCTCCAGTTTCCTTACCC
OsNRAMP5	CAGCAGCAGTAAGAGCAAGATGG	GGGAGGTCGTTGTGGATGAGC
OsACTIN	AGGAAGGCTGGAAGAGGACC	CGGGAAATTGTGAGGGACAT

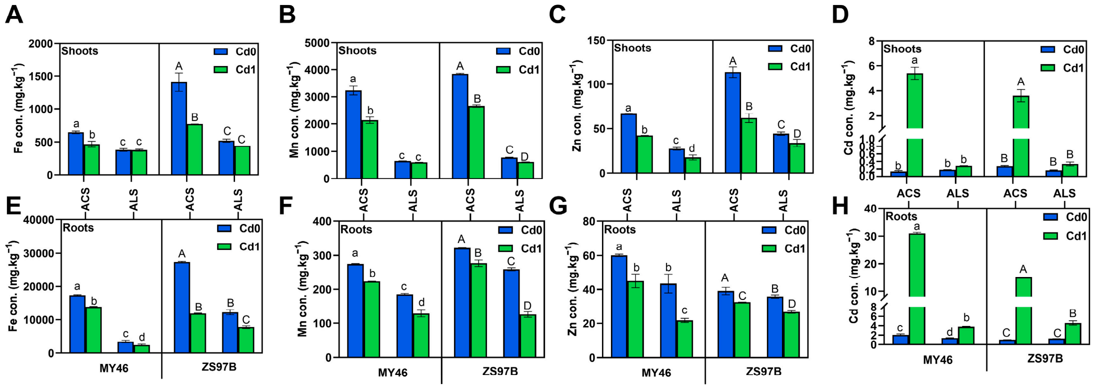

Figure S1. Concentrations of Fe (A, E), Mn (B, F), Zn (C, G), and Cd (D, H) in shoots and roots of MY46 and ZS97B rice plants under varying Cd treatments in acidic and alkaline soils at the maturity stage. ACS, acid soil; ALS, alkaline soil; Cd 0, no Cd stress; Cd 1, add 1 mg/kg Cd stress; Data are means ± SD (n = 3). Mean with different lowercase (MY46) and capital (ZS97B) letters are significantly different (LSD, $p < 0.05$) with regard to treatments.

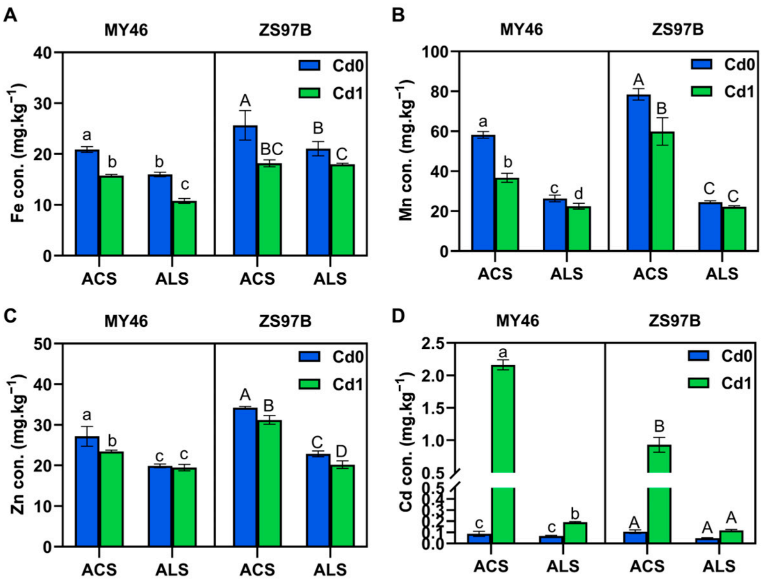

Figure S2. Concentrations of Fe (A), Mn (B), Zn (C), and Cd (D in brown rice of MY46 and ZS97B under varying Cd treatments in acidic and alkaline soils. ACS, acid soil; ALS, alkaline soil; Cd 0, no Cd stress; Cd 1, add 1 mg/kg Cd stress; Data are means ± SD (n = 3). Mean with different lowercase (MY46) and capital (ZS97B) letters are significantly different (LSD, $p < 0.05$) with regard to treatments.

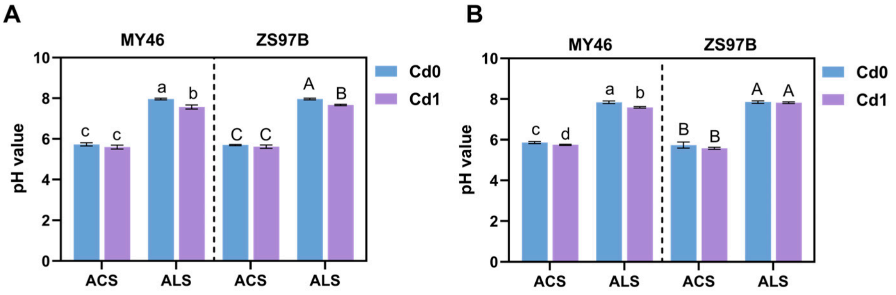

Figure S3. Effects of cadmium stress on soil pH at rice tillering and maturity stages in acidic and alkaline soils. (A) Soil pH at tillering stage of rice; (B) Soil pH at maturity stage of rice. ACS, acidic soil; ALS, alkaline soil; Cd 0, no Cd stress; Cd 1, add 1 mg/kg Cd stress; Data are means ± SD (n = 3). Mean with different lowercase (MY46) and capital (ZS97B) letters are significantly different (LSD, $p < 0.05$) with regard to treatments. The dotted lines separate the two varieties.

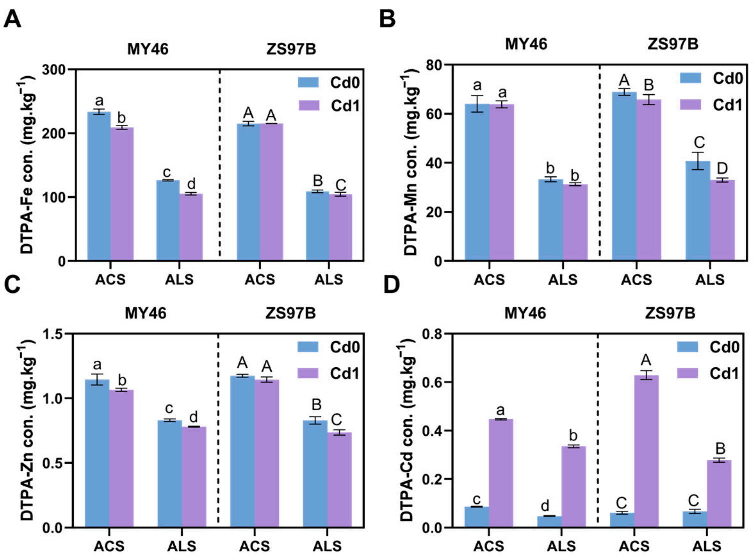

Figure S4. Concentrations of DTPA-extractable Fe, Mn, Zn and Cd in acidic and alkaline soils under varying cadmium stresses during the maturity stage of rice. (A-D) Available contents of Fe, Mn, Zn and Cd were determined by DTPA extractor. ACS, acidic soil; ALS, alkaline soil; Cd 0, no Cd stress; Cd 1, add 1 mg/kg Cd stress; Data are means ± SD (n = 3). Mean with different lowercase (MY46) and capital (ZS97B) letters are significantly different (LSD, p < 0.05) with regard to treatments. The dotted lines separate the two varieties.
